# The disparity in hesitancy toward COVID-19 vaccination between older individuals in nursing homes and those in the community in Taizhou, China

**DOI:** 10.1186/s12877-023-04518-5

**Published:** 2023-12-09

**Authors:** Li Lv, Xu-Dong Wu, Huan-Jun Yan, Shuang-Ying Zhao, Xiao-Dong Zhang, Ke-Lei Zhu

**Affiliations:** 1 The Affiliated People’s Hospital of Ningbo University, Ningbo, 315040 Zhejiang Province China; 2 Hepatopancreatobiliary Surgery, People’s Hospital Affiliated to Ningbo University, Ningbo University, 251 Baizhang East Road, Ningbo, 315040 Zhejiang Province China

**Keywords:** COVID-19, Vaccine hesitancy, Older individuals, Nursing homes, Community

## Abstract

**Purpose:**

Older individuals are priority coronavirus disease 2019 (COVID-19) vaccine recipients. Our aim was to investigate the prevalence of and factors influencing vaccine hesitancy in older individuals living in nursing homes and communities.

**Methods:**

A self-administered COVID-19 vaccine hesitancy survey was conducted from September 2021 to December 2021 among people aged ≥ 60 years in eight nursing homes (382 participants) and the community (112 participants) in Taizhou, China. The response rate was 72.1% (382/530) for older adults in nursing homes and 68.7% (112/163) for older adults in the community.

**Results:**

We found that 58.1% of the older individuals in nursing homes and 36.6% of those in the community were hesitant to receive the COVID-19 vaccine and that there was a statistically significant difference (*P* < 0.001). Multiple logistic regression results indicated that the main factors influencing hesitation among the older individuals in nursing homes were being male (Odds Ratio (OR) = 1.67, 95% Confidence Interval (CI): 1.01–2.76); their cognitive level, including having a high perceived risk of COVID-19 infection (OR = 3.06, 95% CI: 1.73–5.43) or the perception of low vaccine safety (OR = 3.08, 95% CI: 1.545- 6.145); anxiety (OR = 3.43, 95% CI: 1.96–5.99); and no previous influenza vaccination (OR = 1.82, 95% CI: 1.13–2.93); whereas those for older individuals in the community were comorbid chronic diseases (OR = 3.13, 95% CI: 1.11- 8.78) and community workers not recommending the vaccine (OR = 8.223, 95% CI: 1.77–38.27).

**Conclusion:**

The proportion of older individuals in nursing homes who were hesitant to receive the COVID-19 vaccine was significantly higher than for older individuals in the community. Targeted measures should be implemented to reduce vaccine hesitancy and improve vaccination rates in response to the special environment of nursing homes and the characteristics of this population.

## Introduction

The relentless march of the coronavirus disease 2019 (COVID-19) pandemic across the globe has cast a pall, with advanced age emerging as a prominent harbinger of grim tidings. The older demographic lies squarely in the crosshairs of the novel coronavirus (SARS-CoV-2), bearing the brunt of this scourge at disproportionately high rates of grievous affliction, hospitalization, and fatality. This foreboding vulnerability of seniors has become a worrisome preoccupation for myriad scholars [1–5].

Inoculation constitutes a vital bulwark against the predations of COVID-19. Numerous studies have illuminated that COVID-19 vaccines mitigate the dire repercussions of infection amongst older populations, including hospitalization, intensive care admissions, and death, and has a good preventive effect and safety profile [6–17]. Several national vaccination programs have prioritized older individuals as vaccine recipients [2, 5, 18–21].

Table 1 illustrates research on COVID-19 vaccine hesitancy in older adults across varied nations. These studies primarily explore trepidation among community-dwelling seniors or disparate ethnicities [22–44]. However, investigations into such alarm amongst nursing home residents remain scarce. Institutionalized elders and their external counterparts constitute distinct cohorts with divergent backgrounds and lifestyles. Contrasting the vaccination knowledge permeating these elderly groups could unveil variances in perspectives and behaviors between distinct populations. Such comparisons proffer invaluable discernment into the heterogeneity of inoculation intentions, thereby fostering a more profound comprehension of this diversity. Amidst the relentless pandemic, nursing home residents have consistently been deemed an imperiled cluster. However, research into their vaccination inclinations and qualms persists as a relative paucity. Juxtaposing the inoculation status and trepidation of community seniors and institutionalized elders could address this knowledge vacuum, furnishing salient insights to better safeguard the nursing home populace.

**Table 1:** Research overview on vaccine hesitancy among older adults in various study populations

Author	Study design	Study Sample	Setting	Content	Reference
Christian et al.	Cross-sectional	Total:42583	Denmark	The relationship between prayer frequency and vaccine hesitancy	[39]
Sanghavi et al.	Cross-sectional	Total:59	India	The relationship between perceptions of vaccines and vaccine hesitancy	[33]
Tânia et al.	Cross-sectional	Total:602	Portugal	The impact of perceptions, knowledge, and attitudes on vaccine hesitancy	[36]
Divya et al.	Cross-sectional	Total:5784	America	Impact of primary COVID-19 information sources on vaccine hesitancy and uptake among community-dwelling older adults	[25]
Micah et al.	Cross-sectional	Total:6094	Singapore	The impact of information sources on vaccine hesitancy among older adults	[38]
Andaleeb et al.	Cross-sectional	Total:350	Jordan	The impact of vaccine attitudes and cognition on vaccine hesitancy	[22]
Zhang et al.	Cross-sectional	Total:2109	China	Factors influencing vaccine hesitancy among elderly people living alone or with partners	[44]
Judy et al.	Cross-sectional	Total:31	China	Factors influencing vaccine hesitancy at the individual level, social level, etc	[37]
Robbert et al.	Cross-sectiona	Total:23	America	Barriers to COVID-19 vaccination among racial and ethnic minorities and reasons for hesitancy and measures	[28]
Farooq et al.	Case control study	Total:141	Thailand	Using vaccinated people as the case group, a case–control study was used to explore the influencing factors of vaccine hesitancy among the elderly in Muslim communities	[31]
Nandini et al.	Cross-sectional	Total:3804	India	Hesitancy and influencing factors of COVID-19 vaccine booster shots among the elderly in slums and immigrant areas	[35]
Paul et al.	Cross-sectional	Total:370	China	The influencing factors of hesitancy for the second booster dose of the COVID-19 vaccine	[26]
Boaz et al.	Cross-sectiona	Total:400	Israel	Factors influencing self-perception and vaccine hesitancy on vaccination	[24]
Janna et al.	Cross-sectiona	Total:24	America	The impact of gender and race on COVID-19 vaccination in older adults	[34]
Aminath et al.	Cross-sectional	Total:21,663	Singapore	COVID-19 vaccine hesitancy and influencing factors	[29]
Yang et al.	Cross-sectional	Total:1341	China	Reducing drivers of vaccine hesitancy	[42]
Noura et al.	Cross-sectional	Total:1037	Syria	Reasons for vaccine hesitancy among older refugees	[32]
Lu et al.	Cross-sectional	Total:225	China	Link to frailty and vaccine hesitancy in older adults	[41]
Lu et al.	Cross-sectional	Total:725	China	The relationship between cognitive factors and vaccine hesitancy	[45]
Mohammed et al.	Cross-sectional	Total:488	Saudi Arabia	Factors influencing vaccination willingness among community elders	[23]
Marta et al.	Cross-sectional	Total:19	Swiss	Factors responsible for vaccine hesitancy and willingness to be vaccinated	[27]
Anthony et al.	Cross-sectional	Total:400	Ghana	Conspiracy theories, trust in public health information, social capital, and the impact of vaccine hesitancy	[30]
Yuan et al.	Cross-sectional	Total:27	China	Factors influencing vaccine hesitancy in the elderly and strategies to address it	[43]
Wang et al.	Cross-sectional	Total:9890	China	Determinants of COVID-19 vaccination status and hesitancy	[40]

Numerous reports in the literature of outbreaks of COVID-19 in nursing homes showed high rates of infection and mortality [46–54]. Older individuals in nursing homes have a more serious risk of COVID-19 infection compared with the general community of the older individuals, as they are relatively older, often immunocompromised, have more comorbid chronic diseases [55] and more complex conditions, and include a higher number of patients with dementia who have cognitive problems [49]. Nursing homes are high-risk environments for respiratory disease outbreaks, including the transmission of the COVID-19, and the older individuals living in nursing homes are highly vulnerable to such outbreaks, including influenza [56–58]. Nursing homes represent collective residential settings, characterized by communal bathing and restroom facilities, communal dining arrangements, and facets of group communication. Furthermore, a substantial number of elderly individuals grappling with dementia and various cognitive impairments find themselves unable to endure the wearing of masks or the observance of social distancing protocols. Residing within a nursing home setting further necessitates regular interaction with healthcare personnel, thereby heightening the susceptibility to disease transmission [47, 51, 59–61].

This investigation was undertaken with the aim of elucidating the disparities in COVID-19 vaccine hesitancy among elderly denizens residing in nursing homes as compared to their counterparts within the broader community. Our objective was twofold: to scrutinize the determinants underpinning vaccine hesitancy and to enhance the efficacy of vaccination strategies.

## Methods

### Study design and population

We conducted an anonymous cross-sectional and an online, randomly selected survey through WeChat Questionnaire Star (Ranxing Information Technology Co., Ltd., Changsha, Hunan, China), the largest online survey platform in China.

Our target population was adults aged 60 years or older living in eight nursing homes and one community in Taizhou, China. We selected a sample of the largest community and a nursing home in Taizhou, China. They represent community-based older adults and nursing home older adults in this area, respectively. We engaged nursing home staff and family members to assist and explain the questionnaire to older participants with low literacy and limited education, ensuring their meaningful involvement. From September 2021 to December 2021, self-administered questionnaires were answered by scanning a quick response code based on the COVID-19 vaccination status. Sample size determination using the G*Power software (ver. 3.1.9.7; Hein-rich-Heine-Universität Düsseldorf, Düsseldorf, Germany).We used χ^2^ tests = Goodness-of-fit tests: Contingency tables, analysis = A priori: Compute required sample size, Effect size w = 0.5, α err prob = 0.05, power (1-β err prob) = 0.95,Df = 5.The minimum sample size was computed as 80 and the actual power was computed as 0.95.We invited a total of 530 nursing home residents and 163 older individuals living in the community.We excluded those who could not understand the questionnaire information owing to dementia and other cognitive dysfunction. Among them, 49 older individuals were from nursing homes,while 26 were from the community.After further excluding 124 incomplete questionnaires, a total of 382 questionnaires completed by nursing home residents and 112 questionnaires completed by the older individuals living in the community were included in our analysis. The response rate was 72.1% (382/530) for older adults in nursing homes and 68.7% (112/163) for older adults in the community.

This study was approved by the Ethics Committee of Taizhou Hospital, Zhejiang Province, China (approval number: k20210217). All procedures were performed in accordance with the guidelines of our institutional ethics committee and adhered to the principles of the Declaration of Helsinki. All participant information was anonymous. All participants provided informed consent.

### Structured questionnaires

**Fig. 1:**
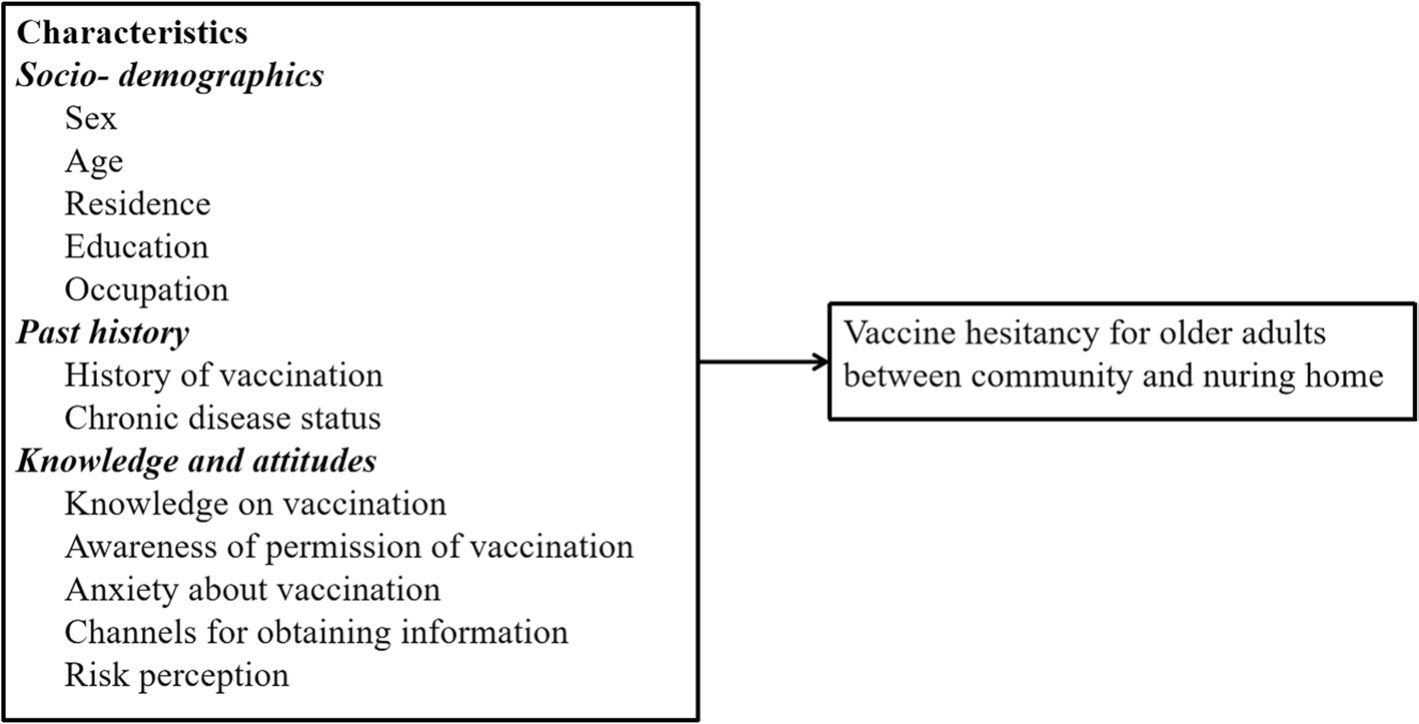
Framework for studying the outcome variables

We designed the questionnaire based on the instructions of the COVID-19 vaccine manufacturers and after consultation with clinical and disease prevention experts. The participants were required to complete the questionnaire by providing information as follows: (1) their basic information, such as age, sex, education level, occupation, and place of residence; (2) their perceived risk of COVID-19; (3) knowledge of the COVID-19 vaccine, access to vaccine information, and attitude toward COVID-19 vaccine; "We assessed the attitudes of older individuals towards COVID-19 vaccination by asking, 'What is your attitude towards receiving the COVID-19 vaccine?' with four response options: 'Willing to receive it as soon as possible,' 'Willing to receive it at a later date,' 'Can choose not to receive it,' and 'Absolutely unwilling to receive it.' The first two options were combined as 'Willing to receive,' and the latter two options were combined as 'Unwilling to receive.'" (4) their history of vaccination, such as their history of vaccine allergy; (5) their chronic disease status, such as hypertension, diabetes, etc., and the disease treatment; (6) their level of anxiety about vaccination; “We assessed anxiety among older individuals regarding COVID-19 vaccination through the following question: “Do you experience anxiety about receiving the vaccine?” Four response options were provided: Very anxious, Anxious, Not anxious, and Not at all anxious. The first two options were combined into Anxious, while the last two options were ultimately merged into Not anxious. “(7) their level of hesitation about receiving the COVID-19 vaccine, and the reasons for their hesitation. “We assessed the hesitancy of elderly individuals towards COVID-19 vaccination using the following question: “Have you ever been hesitant about receiving the vaccine” Four response options were provided: Very hesitant, Hesitant, Not hesitant, and Not at all hesitant. The first two options were combined into Hesitant, while the last two options were ultimately merged into Not hesitant. In addition, various reasons were listed, such as concerns about vaccine side effects, underlying medical conditions, and anticipation of adverse reactions to the vaccine, among others. Fig. 1 depicted the framework for studying the outcome variables.

### Statistical analysis

The primary outcomes of the survey were the points of difference in COVID-19 vaccine hesitancy between the nursing home and community older individuals and the factors influencing that hesitancy. The categorical variables—basic characteristics, including demographic information, knowledge of the vaccine, and attitudes were expressed as counts and percentages. Potential factors influencing COVID-19 hesitancy, such as sex, disease status, knowledge, anxiety, and attitude, were tested using chi-square tests. Age, classified as a continuous variable, was subjected to an independent samples t-test for analysis.To further analyze which factors significantly influenced the COVID-19 vaccine hesitancy of nursing home and community older individuals, we stratified the different factors and analyzed the effect of these factors on hesitation using chi-square tests. The model included variables with *P* < 0.05 in the univariate analysis. A logistic regression model was used to identify factors influencing the hesitancy to receive the COVID-19 vaccine, and the odds ratio (OR) and corresponding 95% confidence interval (CI) were calculated. All data were analyzed by IBM SPSS statistics 26.0 software (SPSS Inc., Chicago, IL, USA). We identified a *P* value of < 0.05 as a statistically significant difference in the target population.

## Results

The participant’s sex, age, education, occupation, place of residence, comorbid chronic diseases, previous influenza vaccination, and level of hesitation about the COVID-19 vaccine are shown in Table 2.

**Table 2:** Basic characteristics of study participants (*n* = 112 + 382)

Independent Variables	Categories	Community	Nursing home	χ2	t	P
Sex	Male	64 (57.10%)	193 (50.50%)	1.520		0.218
	Female	48 (42.90%)	189 (49.50%)			
Age		68.84 ± 8.285	70.15 ± 6.766		1.530	0.128
Education Level	Junior High School and below	111 (99.10%)	240 (62.80%)	55.424		< 0.001
	High school and above	1 (0.90%)	142 (37.20%)			
Occupation	Farmer	30 (26.80%)	219 (57.30%)	32.322		< 0.001
	Worker and others	82 (73.20%)	163 (42.70%)			
Have you received influenza vaccination in the past	No	104 (92.90%)	205 (53.70%)	56.791		< 0.001
	Yes	8 (7.10%)	177 (46.30%)			
Chronic disease status	Two or less	76 (67.90%)	29 (7.60%)	187.935		< 0.001
	More than two	36 (32.10%)	353 (92.40%)			
Hesitation rate	No hesitation	71 (63.40%)	160 (41.90%)	16.093		< 0.001
	Hesitation	41 (36.60%)	222 (58.10%)			

In Table 2, there was no significant difference in age or sex between the two populations. Moreover, more participants living in nursing homes had two or more chronic diseases than those living in the community (92.4% vs. 32.1%, *P* < 0.001). The percentage of nursing home older individuals who were hesitant about the COVID-19 vaccine was much higher than that of community-based older individuals (58.1% vs. 36.6%, *P* =  < 0.001). The factors influencing their hesitation are shown in Table 3.

**Table 3:** Univariate analysis of factors associated with COVID-19 vaccine hesitancy among the older individuals (*n* = 112 + 382)

		**Nursing home**						**Community**					
**Independent Variables**	**Categories**	**Not Hesitant**		**Hesitant**		**χ2**	**P**	**Not Hesitant**		**Hesitant**		**χ2**	**P**
		**N**	**%**	**N**	**%**			**N**	**%**	**N**	**%**		
Total		160	41.90%	222	58.10%			71	63.40%	41	36.60%		
Sex	Male	69	35.80%	124	64.20%	6.029	0.014	44	68.80%	20	31.30%	1.847	0.174
	Female	91	48.10%	98	51.90%			27	56.30%	21	43.80%		
Education Level	Junior High School and below	98	40.80%	142	59.17%	0.293	0.588	70	63.10%	41	36.90%	0.583	0.445
	High School and above	62	43.66%	80	56.30%			1	100.00%	0	0.00%		
Occupation	Farmer	88	40.20%	131	59.82%	0.611	0.434	13	43.30%	17	56.70%	7.105	0.008
	Workers and Others	72	44.17%	91	55.83%			58	70.70%	24	29.30%		
COVID-19 Infection Risk Perception	High	66	28.00%	170	72.00%	49.144	< 0.001	4	80.00%	1	20.00%	0.622	0.430
	Low	94	64.40%	62	35.60%			67	62.60%	40	37.40%		
Chronic disease status	Two or less	102	46.40%	118	53.60%	4.275	0.039	54	71.10%	22	28.90%	5.978	0.014
	More than two	58	35.80%	104	64.20%			17	47.20%	19	52.80%		
Perceived safety of vaccines	Perceived high level of safety	141	44.50%	176	55.50%	5.153	0.023	44	78.60%	12	21.40%	11.119	0.001
	Perceived low level of safety	19	29.20%	46	70.80%			27	48.20%	29	51.80%		
Do you feel anxious when you are not sure whether you can be vaccinated	Anxious	68	27.50%	179	72.50%	59.163	< 0.001						
	Not anxious	92	68.10%	43	31.90%								
Whether to keep an eye on the COVID-19 vaccine news	Yes	101	50.80%	98	49.20%	13.423	< 0.001	39	70.90%	16	29.10%	2.631	0.105
	No	59	32.20%	124	67.80%			32	56.10%	25	43.90%		
Influenza vaccination status	No	73	35.60%	132	64.40%	7.157	0.007	65	62.50%	39	37.50%	0.500	0.479
	Yes	87	49.20%	90	50.80%			6	75.00%	2	25.00%		
The level of the role of medical staff in vaccine recommendations	Less useful	44	27.50%	84	37.80%	4.46	0.035	15	21.10%	27	65.90%	22.185	< 0.001
	Largely useful	116	72.50%	138	62.20%			56	78.90%	14	34.10%		
The level of the role of community staff in vaccine recommendations	Less useful	42	37.50%	70	62.50%	1.252	0.263	8	23.50%	26	76.50%	33.431	< 0.001

Among the older individuals in nursing homes, the more hesitant were men, those with a higher perceived risk of COVID-19 infection, those with a higher number of comorbid chronic diseases, those with a perception regarding the low safety of the vaccine, those who were more anxious about the vaccine, those who were less concerned about keeping up to date with the COVID-19 vaccine via the news media, those who thought medical personnel did not have a useful role in recommending vaccination, and those who had not received influenza vaccines in the past.

Among the older individuals in the community, those more likely to experience vaccine hesitation were farmers, those with a combination of two or more chronic diseases, those who thought the vaccine had a low level of safety, and those who thought neither health workers nor community workers had a useful role in making vaccination recommendations.

The results of the logistic regression analysis are shown in Table 4.

**Table 4:** Multivariate logistic regression analysis of factors associated with COVID-19 vaccine hesitancy in older individuals in nursing homes and the community (*n* = 112 + 382)

Nursing home			
**Variables**	**Categories**	**P**	**OR (95%CI)**
Sex	male vs. female	0.046	1.669 (1.009–2.76)
COVID-19 Infection Risk Perception	high vs. low	< 0.001	3.061 (1.726–5.427)
Perceived safety of vaccines	low vs. high	0.001	3.081 (1.545–6.145)
Chronic disease status	no vs. yes	0.485	1.199 (0.72–1.996)
Do you feel anxious when you are not sure whether you can be vaccinated	yes vs. no	< 0.001	3.428 (1.96–5.995)
The level of the role of medical staff in vaccine recommendations	lesser vs. larger	0.656	1.124 (0.671–1.883)
Whether to keep an eye on the COVID-19 vaccine news	no vs. yes	0.186	1.393 (0.853–2.276)
Influenza vaccination status	no vs. yes	0.013	1.823 (1.133–2.933)
**Community**			
Occupation	farmers vs. others	0.328	1.741 (0.573–5.289)
Chronic disease status	≤ 2 vs. > 2	0.03	3.128 (1.114–8.783)
Perceived safety of vaccines	Low vs. high	0.149	2.156 (0.759–6.126)
The level of the role of medical staff in vaccine recommendations	lesser vs. larger	0.242	3.197 (0.455–22.337)
The level of the role of colleagues, friends and neighbors in vaccine recommendations	lesser vs. larger	0.337	2.692 (0.356–20.328
The level of the role of community staff in vaccine recommendations	lesser vs. larger	0.007	8.223 (1.767–38.271)

In nursing homes, the significant factors affecting vaccine hesitation were being male, having a higher perceived risk of COVID-19 infection, believing the vaccine to be unsafe, being anxious about the vaccine, and having had no previous influenza vaccination.

Among the older individuals in the community, the significant factors affecting vaccine hesitation were perceiving low vaccine safety and the lesser role of community workers in recommending vaccinations.

## Discussion

SARS-CoV-2 has precipitated a significant toll in terms of mortality within the global older patient demographic. Advanced age, coupled with underlying medical conditions, emerges as a pivotal determinant accentuating susceptibility to COVID-19 [61–63]. Nursing homes find themselves acutely impacted by the COVID-19 pandemic, with the older residents therein navigating an elevated susceptibility to contracting COVID-19 [64, 65]. Vaccine hesitancy is a complex public health issue, [66] and various studies have shown that older individuals have a higher rate of COVID-19 vaccine hesitancy [67–71] compared with younger people.

Our study found that the percentage of vaccine hesitancy among nursing home older individuals was significantly higher than that among the older individuals living in the community (58.1% vs. 36.6%).

Further analysis illuminated that vaccine hesitancy amongst nursing home residents stems chiefly from personal perceptions, including apprehensions about COVID-19 vaccine safety, perceived susceptibility to COVID-19 infection, anxiety towards the vaccine, gender, and past influenza vaccination behavior. These subjective factors constitute the primary determinants of inoculation reluctance within this institutionalized population.

In contrast, vaccine hesitancy among community-dwelling older individuals was chiefly driven by health status, particularly the burden of chronic comorbidities, and the role of community staff in advocating for vaccination. These objective factors constituted the primary determinants of inoculation reluctance within this non-institutionalized population.

Elevated perceptions of risk and a heightened level of confidence in the COVID-19 vaccine have been established as pivotal factors in mitigating vaccine hesitancy [72–76]. In contrast, within the purview of our inquiry, an elevated perception of the susceptibility to COVID-19 infection exhibited an association with vaccine hesitancy. Upon meticulous review of the extant literature, we unearthed an incipient connection between heightened perceived risk and anxiety, as well as a substantive nexus linking vaccine hesitancy to psychological variables, foremost among them being anxiety [, 77]. Several inquiries have demonstrated that diminished levels of anxiety exert a mitigating influence on vaccine hesitancy, even in instances where there is an elevation in the perception of risk [78]. In our investigation, we also discerned that individuals who harbored a heightened perception of their susceptibility to COVID-19 infection manifested greater trepidation regarding the prospect of vaccination. The framework of risk perception elucidates that individuals harboring a heightened perception of risk may exhibit diminished motivation, rendering them less inclined to partake in self-preservative health practices, thereby contributing to a surge in vaccine hesitancy rates. (Rimal&Real, 2003) [78].

Another salient factor influencing vaccine hesitancy among nursing home residents was prior influenza vaccination behavior, with those previously inoculated against influenza exhibiting less reluctance towards COVID-19 vaccines. Past receptivity to influenza jabs can shape general vaccine attitudes, thus also informing COVID-19 vaccine perspectives within this institutionalized elderly population [79, 80]. Some studies have shown a strong correlation between the previous hesitation toward the influenza vaccine and hesitation toward the COVID-19 vaccine [67, 81, 82].

Additional factors contributing to hesitancy among the elderly residents of nursing homes encompassed a heightened incidence of hesitation observed among males as compared to their female counterparts, a pattern congruent with extant literature findings [83]. The advocacy for vaccine dispensation by healthcare professionals has assumed a pivotal role in assuaging hesitancy among older denizens residing in nursing homes. Within these establishments, the older populace routinely engages with healthcare practitioners, from whom they primarily glean information [53, 49]. Healthcare professionals persist as a paragon of trustworthiness, adept in furnishing counsel and insights to elderly individuals who harbor reservations about COVID-19 vaccine inoculation, thereby augmenting their receptivity to persuasion.

With regard to the older members of the community, their individual health status wielded a more substantial influence on vaccine hesitancy. Specifically, a higher burden of comorbid chronic illnesses was associated with an elevated level of hesitancy. Existing literature posits that individuals in good health tend to exhibit lesser reluctance towards vaccine acceptance in contrast to those afflicted with comorbid conditions. Furthermore, it underscores the role of chronic ailments in both exacerbating the prevalence of vaccine hesitancy and shaping vaccination uptake rates" [74, 84]. The advocacy for vaccine administration by community personnel has assumed a pivotal role in mitigating hesitancy among older inhabitants of the community. Empirical evidence has elucidated that populations residing within communal environs are notably amenable to peer influence concerning vaccine hesitancy. In the context of community settings, older individuals engage in more frequent interactions with community personnel, rendering them more susceptible to the sway of their counsel and guidance [85–87].

Varied perspectives necessitate multifaceted interventions, tailored to address the distinct determinants of vaccine hesitancy observed within the disparate populations of nursing home residents and community-dwelling older individuals. In the context of nursing homes, targeted initiatives should encompass bolstered education for the apprehensive cohort, heightened dissemination of COVID-19 and vaccine-related information among the anxious or skeptical, meticulous oversight of vaccination protocols, and comprehensive guidance for healthcare personnel. Conversely, within the community-dwelling elderly demographic, the focus ought to be directed towards enhancing the management of chronic ailments, enshrining a regimen of regular screening and health education by community physicians, thereby augmenting both physical well-being and quality of life among this population.

This study is not without its inherent limitations. Firstly, our survey was confined to specific communities and institutions within a singular region, thereby constraining the generalizability of the sampled population. Secondly, data collection relied on smartphone-based software, potentially excluding older individuals devoid of such technology, which could introduce bias. Thirdly, due to the modest sample size, conducting separate analyses for the two distinct groups may have reduced statistical robustness. Fourthly, while this article incorporates two distinct questionnaires, it is noteworthy that the questionnaire administered to community-dwelling elderly participants did not encompass inquiries regarding anxiety. Consequently, the capacity to analyze the nexus between vaccine hesitancy and anxiety among the elderly in the community remains unattainable. Lastly, it is imperative to acknowledge the intricate nature of factors contributing to vaccine hesitancy; thus, this study may not have exhaustively identified all potential determinants associated with hesitancy.

## Conclusion

Our investigation revealed a substantial disparity in COVID-19 vaccine hesitancy between the elderly denizens of nursing homes and their counterparts dwelling within the community.

The factors influencing COVID-19 vaccine hesitancy were different in the two populations; the nursing home older individuals were mainly influenced by their perception of the COVID-19 vaccine, whereas the factors for the older individuals living in the community were mainly associated with their own health status.

Hence, in our endeavor to safeguard the health of our elderly populace by augmenting COVID-19 vaccination rates, it is imperative to discern the distinctive attributes of older individuals residing in nursing homes and the community. This discernment shall enable us to furnish bespoke, precise interventions and educational initiatives, ensuring the most efficacious mitigation of vaccine hesitancy.

## Data Availability

Some or all data, models, or code generated or used during the study are available from the corresponding author by request.
